# Improvement of Water Vapor Permeability in Polypropylene Composite Films by the Synergy of Carbon Nanotubes and β-Nucleating Agents

**DOI:** 10.3390/polym15224432

**Published:** 2023-11-16

**Authors:** Glykeria A. Visvini, Georgios N. Mathioudakis, Amaia Soto Beobide, Zoi Piperigkou, Aris E. Giannakas, Stavros Messaritakis, Giannis Sotiriou, George A. Voyiatzis

**Affiliations:** 1 Foundation for Research and Technology-Hellas (FORTH), Institute of Chemical Engineering Sciences (ICE-HT), Stadiou Str., 265 04 Rio-Patras, Greece; gvisvini@iceht.forth.gr (G.A.V.); mathioy@iceht.forth.gr (G.N.M.); asoto@iceht.forth.gr (A.S.B.); 2 Department of Physics, University of Patras, 265 04 Rio-Patras, Greece; 3 Department of Materials Science, University of Patras, 265 04 Rio-Patras, Greece; 4 Laboratory of Biochemistry, Department of Chemistry, Biochemical Analysis & Matrix Pathobiology Research Group University of Patras, 265 04 Rio-Patras, Greece; zoipip@upatras.gr; 5 Department of Food Science & Technology, University of Patras, 301 00 Agrinio, Greece; agiannakas@upatras.gr; 6 Plastika Kritis S.A., Industrial Area of Heraklion, R Street, Heraklion, 714 08 Crete, Greece; messaritakis@plastikakritis.com; 7 Thrace Polyfilms S.A., Industrial Area Xanthi, 671 00 Xanthi, Greece; gsotiriou@thraceplastics.gr

**Keywords:** nanocomposites, carbon nanotubes, nucleating agent, breathability, polypropylene

## Abstract

A notable application of polymeric nanocomposites is the design of water vapor permeable (WVP) membranes. “Breathable” membranes can be created by the incorporation of micro/nanofillers, such as CaCO_3_, that interrupt the continuity of the polymeric phase and when subjected to additional uniaxial or biaxial stretching this process leads to the formation of micro/nanoporous structures. Among the candidate nanofillers, carbon nanotubes (CNTs) have demonstrated excellent intrinsic WVP properties. In this study, chemically modified MWCNTs with oligo olefin-type groups (MWCNT-g-PP) are incorporated by melt processes into a PP matrix; a β-nucleating agent (β-ΝA) is also added. The crystallization behavior of the nanocomposite films is evaluated by differential scanning calorimetry (DSC) and X-ray diffraction (XRD). The WVP performance of the films is assessed via the “wet” cup method. The nanohybrid systems, incorporating both MWCNT-g-PP and β-NA, exhibit enhanced WVP compared to films containing only MWCNT-g-PP or β-NA. This improvement can be attributed to the significant increase in the growth of α-type crystals taking place at the edges of the CNTs. This increased crystal growth exerts a form of stress on the metastable β-phase, thereby expanding the initial microporosity. In parallel, the coexistence of the inherently water vapor-permeable CNTs, further enhances the water vapor permeability reaching a specific water vapor transmission rate (Sp.WVTR) of 5500 μm.g/m^2^.day in the hybrid composite compared to 1000 μm.g/m^2^.day in neat PP. Notably, the functionalized MWCNT-g-PP used as nanofiller in the preparation of the “breathable” PP films demonstrated no noteworthy cytotoxicity levels within the low concentration range used, an important factor in terms of sustainability.

## 1. Introduction

The development of polymer nanocomposites has attracted intense interest in the last decades and can be regarded as a significant technological advancement in the plastics industry due to their unique properties, which can be tuned by controlling the type or/and the concentration of the nano-inclusions. Polymer nanocomposites are high-performance materials and possess numerous favourable features, such as high mechanical strength, low weight, ease of processing and low matching cost [1]. These improved properties, in comparison to the common macro/micro-composite materials, make them ideal materials for various engineering applications. An essential use case involves polymeric membranes that facilitate the transport of air and water vapor. The COVID-19 pandemic has decisively highlighted the usefulness of such antimicrobial membranes in facemasks. Another common application refers in medical and hygiene products and in food packaging. However, in other sections, such as in construction materials, the water vapor transport is the most important feature. In this case, such a membrane can minimize both the risk of condensation within the building fabric by reducing the transport of water vapor through the building elements and, at the same time, the convective heat losses contributing to the improved energy efficiency of the building. According to this context, breathability is defined as the ability of some membranes to allow the diffusion of water vapors through them and at the same time to prevent the penetration of water in liquid state [2,3]. 

Polymeric films enabling water vapor permeability, beyond the non-woven SMS (spunbond-meltblown-spunbond) membranes (i.e., facemasks), can be designed by using micro/nano-fillers, which have the ability to interrupt the continuity of the polymer phase generating micro/nano-porous structures by further uniaxial/biaxial stretching [2]. Specifically, the incorporation of inorganic fillers in polyolefins, can improve the breathability characteristics as well as the mechanical properties of the composite polymeric membranes [4,5,6,7].

Carbon nanotubes (CNTs) have undoubtedly attracted extensive attention, due to their exceptional mechanical, thermal, electrical, optical, and chemical properties [8,9,10,11,12,13], making them promising materials in various fields. Another approach, which has attracted particular interest, is the use of CNTs to control water transport in membranes [14,15], owing to their intrinsic relevant properties [16,17]. Water flow through membranes composed of an array of aligned CNTs is reported to be orders of magnitude faster than that predicted from the conventional fluid flow theory. This feature is attributed to CNTs’ high aspect ratio (high length-to-diameter ratios) and their frictionless, smooth, chemically inert graphitic walls that together with their internal diameters in the nanoscale [18] represent an excellent candidate for both high flow rate and high rejection of low molecular weight organic molecules [19], properties that have conventionally been complementary. Kannan et al., in 2013, reviewed and compared all experimental and theoretical studies on the water flow in CNTs, published since 2004 and suggested a 3 to 2 orders of magnitude water flow enhancement through CNTs with internal diameters of 1.6 to 6.5 nm, respectively. In the same context, they also suggested that as the diameter of the tube increases above 10 nm, the flow rate slowly approaches the classical Navier-Stokes prediction with no significant enhancement [20]. C. Tang et al. observed the effect of MWCNT content on the water transport behavior of porous chitosan membranes. The water transport, constant in composite membranes up to a critical MWCNT content (5% by weight), was increasing at higher concentrations of MWCNT. The water flux of composite membrane with 10 wt.% MWCNTs (128.1 L/m^2^h) was measured 4.6 times that of neat one (27.6 L/m^2^h) [21]. An important aspect to be addressed with CNTs is their tendency to form aggregates or bundles, due to strong van der Waals forces, which results in their agglomeration and inhomogeneous dispersion problems during the development of relevant polymer nanocomposites. To avoid this problem, chemical modification of CNTs with compatible to the corresponding polymeric matrix groups is attempted [22,23,24]. 

PP is a thermoplastic polymer, which has been extensively studied in a variety of applications, including films [25,26]. It possesses an attractive combination of easy processability, low cost and versatile properties, such as low density, high chemical resistance and high degree of crystallinity [27]. The crystalline structure of PP has an important effect on its final properties. Specifically, isotactic PP (i-PP) is a semi-crystalline polymer that exhibits a very interesting polymorphic behavior, depending on the polymerization procedure, thermal history and use of different nucleating agents. There are four different crystalline structures of i-PP, namely monoclinic α-form, hexagonal β-form, orthorhombic γ-form as well as the smectic phase [28]. Among these crystalline structures, the monoclinic α-form is the most stable modification and is found in all solution-crystallized PP samples [28,29,30,31,32]. The metastable hexagonal β-form has the tendency to transform to the α-form when exposed to mechanical or thermal stress [33,34,35,36]. Specifically, the β-phase, being controlled by the thermal history, may re-crystallize to α-phase upon heating. In this context, the β-form can be produced under special crystallization conditions or in the presence of selective β-NA [28,29,30,37,38,39,40]. The addition of a β-NA is the most effective and practical way for the preparation of β-nucleated PP [41]. The orthorhombic γ-form can be developed in the presence of chain defects, which limit the isotactic sequences, in degraded, low-molecular weight PP or during crystallization at elevated pressure [42,43]. Finally, the smectic phase can be considered as a fourth polymorph, which is mainly formed upon supercooling of the isotropic melt. 

Many authors have devoted their research to clarifying the pore formation mechanism of β-nucleated PP during stretching [44,45,46,47,48]. It has been proven that the growth of β-phase in PP, in the presence of a β-NA in conjunction with stretching, can lead to the preparation of microporous membranes that exhibit enhanced water vapor permeability [49,50]. 

On the other hand, CNTs have the tendency to act as α-nucleators when dispersed in PP [51] and their nanoscale dimensions allow for the creation of tortuous pathways within the material, facilitating the diffusion of water vapor. 

Polypropylene (PP), as one of the commodity plastics, is candidate as polymer matrix for nanocomposites. To the best of our knowledge, this is the first work dealing with water vapor transport through melt-mixed polypropylene membranes containing functionalized MWCNTs (MWCNT-g-PP) with varying β-NA contents. For the preparation of breathable PP composites and considering the competition between α- and β-modification growths, the β-NA/CNTs mass ratio should be controlled at an optimal content. The latter was already reported as able to maintain a corresponding percentage of the β-crystalline phase [52]. 

The main scope of present work is the cross-examination of the effect of both β-ΝA and CNTs on the water vapor transmission rate properties of i-PP. Key-role parameters, such as the filler loading being related to the crystallization behavior and the structural properties of PP, are also investigated. The permeability property of semicrystalline polymers is closely related to the crystal composition as well as the crystal morphology [53]. The potential cytotoxic effects of the specific CNTs were examined following the in vitro exposure to broad concentration range of CNTs, mainly considering the possible infection of industrial workers handling these nanomaterials or even the CNTs eventual release from construction materials during their use. The findings of this study will be useful for the development of an alternative pathway in order to prepare PP-based composites with desirable water vapor transmission rate properties and an expected improved performance/cost ratio over the existing stretched reference PP/CaCO_3_ composite benchmark membranes, through the simultaneous addition of carbon nanotubes and a β-ΝA. These newly produced PP/β-NA/MWCNT-g-PP breathable membranes could be suitable for two specific applications: either (a) as roofing membranes, which would allow water vapor to diffuse through them and avoid moisture condensation; and/or (b) as vapor control layers, which will have the ability to reduce water vapor transport through a structural building element and minimize convective heat loss.

## 2. Materials and Methods

### 2.1. Materials

Amino surface modified MWCNTs (MWCNT-NH_2_, CAS: 99685-96-8, outer diameter < 20 nm and length ~ 1–12 μm) were purchased from Cheaptubes Inc., Grafton, VT, USA. The polypropylene grafted maleic anhydride (PP-g-MA, CAS: 25722-45-6) was purchased from Sigma Aldrich (St. Louis, MO, USA). The powdered form of polypropylene (ECOLEN^®^ HZ40S polypropylene homopolymer) was a product of the Hellenic Petroleum S.A. (Athens, Greece). The β-nucleating agent (β-ΝA) used for the preparation of the composites, was provided by Plastika Kritis S.A. (Crete, Greece) as master batch of PP, coded KRITILEN NC 191 (PP/10%β-NA).

For cytotoxicity tests the following biochemical and reagents where used: Dulbecco’s minimal essential medium (DMEM), fetal bovine serum (FBS), sodium py-ruvate, L-glutamine, penicillin, streptomycin, amphotericin B, and gentamycin were all obtained from Biosera LTD (Cholet, France). Pluronic F-127 was purchased from Sigma Aldrich (St. Louis, MO, USA). All other chemicals used were of the best commercially available grade. 

### 2.2. Membrane Preparation

For the direct grafting of polypropylene chains onto the MWCNT surface (MWCNT-g-PP), polypropylene grafted maleic anhydride (PP-g-MA) reacted with the amino surface modified MWCNTs (MWCNT-NH_2_) (ratio 5:2) in a homemade batch mixer at 220 °C and 40 rpm for 30 min [17]. This was followed by grinding in a planetary ball mill. The PP polymer chains grafted onto MWCNTs, MWCNT-g-PP, facilitate a more homogeneous dispersion of the CNTs in the polymer matrix. Nanocomposite membranes containing PP with functionalized MWCNTs (MWCNT-g-PP 0.5, 1.5, 3, 4, and 6 wt.%.) and β-NA (0.3, 1, and 4 wt.%) were prepared by melt mixing with the appropriate amounts of powdered PP (ECOLEN^®^ HZ40S homopolymer) in a twin-screw extruder (Thermoscientific-minilab twin extruder HAAΚΕ MINILAB II with conical screws, ANTISEL, S.A., Athens, Greece) at 200 °C and screw speed of 100 rpm for 3 min. The obtained after the extrusion process composite pellets were transformed into films by using a hydraulic press with heated platens and heat-pressing approximately at 200 °C and 80 bars. The thickness of the obtained films thus obtained was around 100 μm measured with a Starrett F2730IQ micrometer (approximately 10 points were measured and an average thickness was subsequently calculated). Representative SEM images of films after a cryogenic cross-section fracture showing the dispersion of MWCNTs are shown in the Supplementary Information (Figure S1).

Two different cooling rates from the melt were followed to obtain the polymeric membranes: (a) quenching in ice water and (b) annealing at 130 °C for 30 min It is noteworthy that the actual MWCNT loading is lower than the nominal values since the MWCNT content in the MWCNT-g-PP master batch was estimated to be 40% by weight using thermogravimetric analysis (TGA) [17].

## 3. Experimental Techniques

### 3.1. Characterization Techniques

Differential scanning calorimetry (DSC) measurements were carried out on a Q100 unit (ΤA Instruments, New Castle, DE, USA) equipped with a liquid N_2_ cooling system. For the study of melting and crystallization behavior of net polymer and nanocomposites containing CNTs and/or β-NA, the samples (6 mg) were heated up to 200 °C with a heating rate of 10 °C min^−1^. All measurements were performed in a nitrogen atmosphere (50 mL/min). Data were obtained from the first heating cycle that refer to the actual state of the prepared composites. The degree of crystallinity of PP composites is calculated from the melting thermographs by the following equation

$$X_{C}\%=\frac{{\Delta H}_{m}}{{\Delta H}_{m\;}^{ο}\;\left(1-\varphi \right)}\times 100\%$$

where ΔH_m_ and ${\Delta H}_{m\;}^{ο}$ are, respectively, the evaluated specific and the standard fusion heats of either the α- or the β-phase; 178 J/g and 170 J/gr are used as standard fusion heats for 100% crystalline PP in α- and β-phase, respectively [54]. Finally, φ refers to the weight fraction of the fillers.

X-ray diffraction (XRD) was used to identify the crystalline phase of all composites listed in Table 1. XRD measurements were carried out with a Bruker D8 Advance diffractometer equipped by a Cu lamp (λCuKa =1.54046 Å) at a scanning rate 0.5°/min over a range 5°–30° (2θ). Temperature dependence XRD measurements (RT-140 °C) were carried out the same XRD equipped with the XRK900 reactor chamber (Anton Paar GmBH).

The “wet” cup method, described by ASTM E 96-95, was used to measure the water vapor transmission rate (WVTR) through the composite membranes. According to the terminology used by ASTM, the WVTR is defined as the steady-state water vapor flow per time unit through a unit area of a body, occurring normal to its parallel surfaces, under specific controlled conditions of temperature and humidity at each of the two surfaces of the membrane. According to this method, an acetal dish filled with distilled water and tightly covered by the examined membrane is placed in a homemade chamber [13] at controlled conditions of 27 °C and 21% relative humidity (RH). A least-squares regression analysis of the change in mass as a function of time is used to determine the rate of water vapor transmission at steady state. WVTR was calculated from the steady-state region according to equations already reported [17,55,56] and shown in the SI.

### 3.2. In Vitro Cytotoxicity Studies

#### 3.2.1. Cell Cultures and Conditions 

Human lung adenocarcinoma A549 cell line was obtained from the American Type Culture Collection (ATCC, Baltimore, MD, USA) and routinely cultured as monolayers at 37 °C in a humidified atmosphere of 5% (*v*/*v*) CO_2_ and 95% air. Cells were grown in complete DMEM culture medium supplemented with 10% FBS, a cocktail of antimicrobial agents (100 IU/mL penicillin, 100 mg/mL streptomycin, 10 mg/mL gentamicin sulphate and 2.5 mg/mL amphotericin B), 2 mM L-glutamine, and 1 mM sodium pyruvate. Cells were harvested by trypsinisation with 0.05% (*w*/*v*) trypsin in PBS containing 0.02% (*w*/*v*) Na_2_EDTA. All experiments were conducted in serum-free conditions (i.e., 0% FBS). MWCNT-NH_2_ and MWCNT-g-PP were dispersed in 0.1% Pluronic F-127 (PF-127), a non-ionic copolymer surfactant, sonicated in water bath for 15 min in RT, and filtered prior to cell treatment. The respective dilutions of the tested MWCNTs, according to each experimental design were performed in DMEM 0% FBS in a working concentration range 500, 200, 50, 10 and 1 ug/mL.

#### 3.2.2. Phase-Contrast Microscopy

A549 cells were seeded in 24-well plate and incubated for 24 h in complete medium, followed by 16 h starvation (0% FBS). The medium was then replaced with a serum-free medium containing each MWCNT in the respective dilution, and cells were incubated for 72 h in order to determine the effects of long-term exposure on cell morphological characteristics. Photographs were captured utilizing a color digital camera (CMOS) on a phase contrast microscope (OLYMPUS CKX41, QImaging Micro Publisher 3.3RTV) through 10× and 40× objective to monitor cell morphology.

#### 3.2.3. MTT Cell Viability Assay

A549 cells were seeded in 96-well cell culture plates and grown to 60–70% confluency, followed by a 16 h starvation in serum-free medium, prior to treatment with MWCNTs in the concentration range 1–1000 μg/mL for 24 h (short-term effect) and 72 h (long-term effect). To evaluate the effects on cell viability through mitochondrial dehydrogenase activity, MTT [3-(4,5-dimethylthiazol-2-yl)-2,5-diphenyltetrazolium bromide] was added to each well in an amount of 10% in the culture media volume for 4 h at 37 °C and the formazan crystals were dissolved through thorough pipetting. The spectrophotometric measure of cells’ viability was performed in a Tecan spectrophotometer at an absorbance wavelength of 570 nm (reference wavelength at 650 nm), according to the manufacturer’s instructions.

#### 3.2.4. Statistical Analysis

For each assay, three individual experiments were conducted. Data in diagrams are expressed as mean ± standard deviation (SD). Statistically significant differences were evaluated using one-way ANOVA, followed by Tukey’s post hoc test. Statistical analyses and graphs were made using GraphPad Prism 9 (GraphPad Software, San Diego, CA, USA). Statistically significant differences are indicated by asterisks: * (*p* ≤ 0.05), ** (*p* < 0.01), compared with untreated (control) cells. Non-statistically significant comparisons (*p* > 0.05) are not displayed.

## 4. Results and Discussion

### 4.1. Crystallization Behavior of PP Composite Films

A decisive parameter for the composite films that are candidates as water vapor permeable membranes is their crystallization behavior. In this context, differential scanning calorimetry (DSC) and x-ray diffraction (XRD) techniques were used cooperatively for the determination of the characteristics of the crystal structures existing in the composites.

#### 4.1.1. Influence of MWCNT Loading

**Figure 1:**
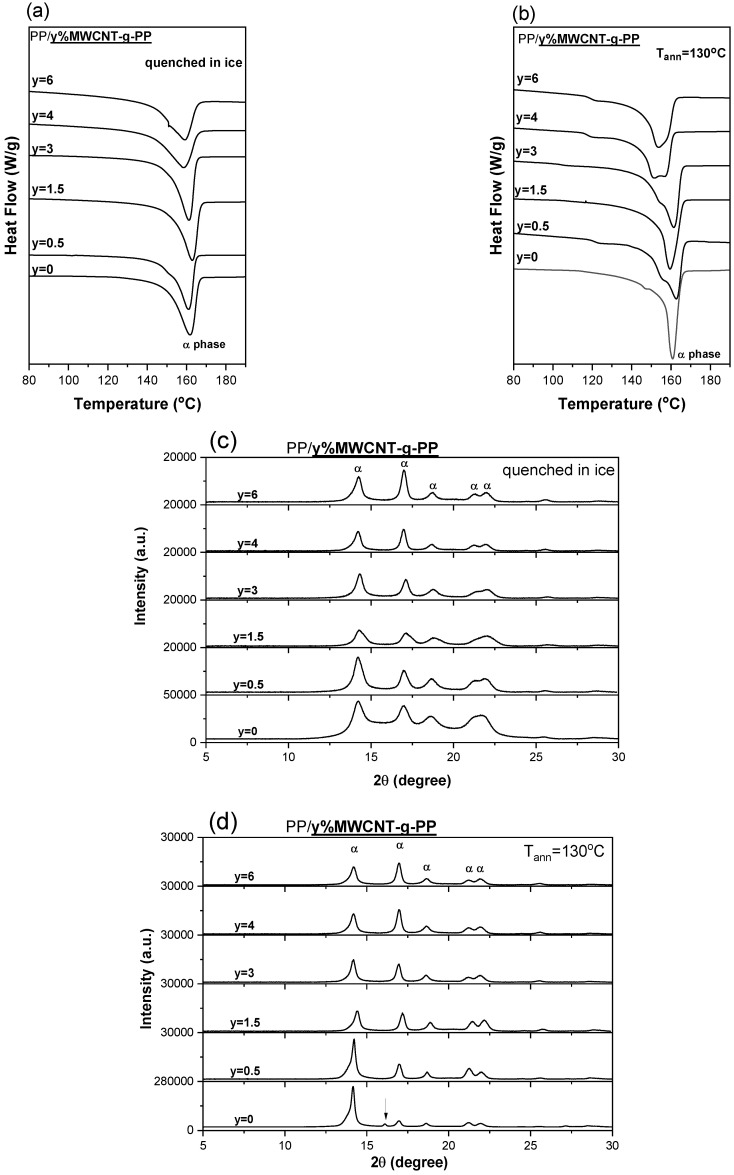
DSC thermographs of composite films PP/MWCNT-g-PP (0, 0.5, 1.5, 3, 4 and 6 wt.%) quenched in ice water from melt (**a**) and annealed at 130 °C (**b**), XRD patterns for these composite films (**c**,**d**).

DSC thermographs of composite films PP/MWCNT-g-PP (0, 0.5, 1.5, 3, 4 and 6 wt.%) are shown in Figure 1a,b. The procedure involved heating from −20 °C to 200 °C at a heating rate of 10 °C min^−1^. Multiple-melting behavior is generally assumed to result from polymorphism, from the successive melting of crystal populations with distinct degrees of perfection. Interestingly is to observe that PP/MWCNT-g-PP composites quenched in ice present only one endothermic melting peak associated with the α-crystalline phase of PP (melting point T_m_~160 °C) [17], which becomes quite broader with MWCNTs loading indicating a wider crystallite size distribution. For the samples crystallized at 130 °C, a diffused shoulder at around 155 °C prior to the melting peak appears; such results may indicate a double melting behavior of PP. The broadening or the shoulder appearance in the melting or the double-melting peaks can be attributed to the imperfect α-crystals, which are melted, recrystallized and then melted again [57]. This indicates that the nanotubes act as heterogeneous crystal α-nucleating agents for the PP even at relatively low content (less than 1 wt.%) and some of the relevant crystal structures being of inferior perfection melt at lower temperatures. From the values presented in Table 2, it is perceived that crystallinity increases with annealing, as expected. However, there is not a clear trend of crystallinity increase/decrease with CNTs loading.

The corresponding crystallization phases were recorded in the XRD diagrams (Figure 1c,d). All samples, independently of thermal history (either quenched from melt or annealed) exhibited five diffraction peaks at 2θ = 14.2°, 17°, 18.7°, 21.4° and 22°, which correspond to the respective crystalline lattice planes, α(110), α(040), α(130), α(111) and α(131) + (041), of the α-crystalline phase of the PP [54]. The diffractograms corresponding to the different composites are quite similar. Some differences, however, are observed between samples quenched from melt or annealed. In the former case, well-define peaks are observed as the carbon nanotube loading increases; this is an indication of particle size increase. On the other hand, for samples annealed at 130 °C, the relative intensity of the peaks at 14.2° and 17° changes with MWCNTs loading due most probably to the orientation of PP crystallites occurring in the presence of fillers with a high aspect ratio [58]. It is also mentioned that the neat PP sample presents a very small diffraction peak at 16° attributed to β-phase crystallites in PP (this peak is indicated with an arrow in Figure 1d). The annealing helped to the development of a small portion of β-phase. On the other hand, it is well-documented that MWCNTs serve as α-nucleating agents for PP. Hence, it is clear that the introduction of CNTs impeded the growth of the metastable β-crystalline phase, in favor of the α-phase promoted by the presence of CNTs.

#### 4.1.2. Influence of β-ΝA Loading

The β crystalline phase of isotactic polypropylene (i-PP), which sometimes survives even in stretched films, has already been associated with the development of microporosity. One way to grow the β crystalline phase in i-PP is by adding a β-nucleating agent (NA). In this context, an analogous study was also carried out for the composites, in which different percentages of β-ΝA were added, a typical material for promoting the growth of β-phase in PP, anticipating that its metastable structure will promote the formation of microporous membranes with subsequently enhanced water vapor permeability capacity. Therefore, the characterization of the crystal structures could indicate the different phases existing in the composites and how the β-ΝA percentage can affect their crystallinity. 

**Figure 2:**
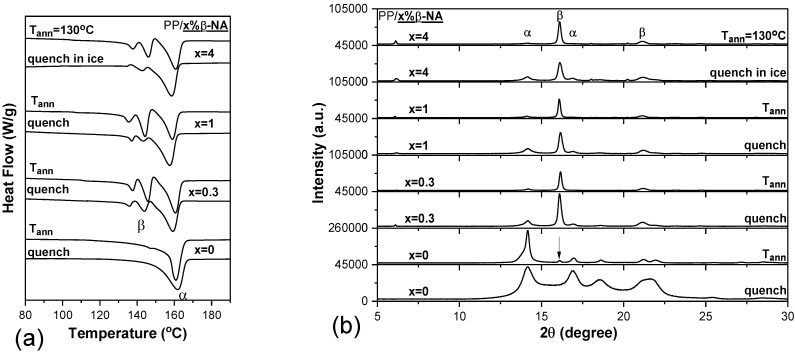
(**a**) DSC thermographs of pure PP and PP/ (0.3, 1 and 4 wt.%) β-NA films (quenched in ice water from melt and crystallized at 130 °C), (**b**) XRD patterns for the same films.

DSC thermographs of neat PP and PP/β-NA films are shown in Figure 2a. In pure PP films, there is only one main melting peak, which is located at ~160 °C and is attributed to the melting of α-crystals of PP, as mentioned above. In the case of PP/β-NA films, apart from the melting peak of the α-phase, two new peaks are observed and are attributed to the characteristic melting peaks of the PP β-phase (β_1_ = 138 °C and β_2_ = 145 °C) [50]. These two peaks appear to be more enhanced in the films subjected to isothermal crystallization (annealing) in an oven at 130 °C. Therefore, the addition of β-nucleator, promotes the β crystalline phase in PP, which is favored in films that have been annealed. The degree of crystallinity of PP composites (X_α_% and X_β_%) is calculated from equation (1), where $\Delta H_{m}^{ο}$ is the standard fusion heat of either α-phase or β-phase PP crystals [59]. The β-NA loading promotes crystallinity of the β-phase (X_β_%). Additionally, this percentage also increases with the annealing process, as shown in Table 3. 

Characteristic XRD reflections diagrams are presented in Figure 2b. It is evident that for all samples loaded with β-NA, apart from the diffraction peaks attributed to the characteristic α phase, the two prominent diffraction peaks at 2θ= 16° and 21°, attributed to the characteristic β phase, appear which correspond to the planes crystal lattice β(300) and β(301) [54,60] of PP. These peaks are even enhanced in the composite films submitted to isothermal crystallization at 130 °C, in agreement with the DSC thermographs. In PP samples loaded with β-NA, it seems that the β-modification exhibits a faster growth rate compared to the α-phase, since the X-ray diffraction (XRD) patterns of the α-crystallites become entirely obscured. 

#### 4.1.3. Influence of the Loading of Both β-NA and MWCNTs

**Figure 3:**
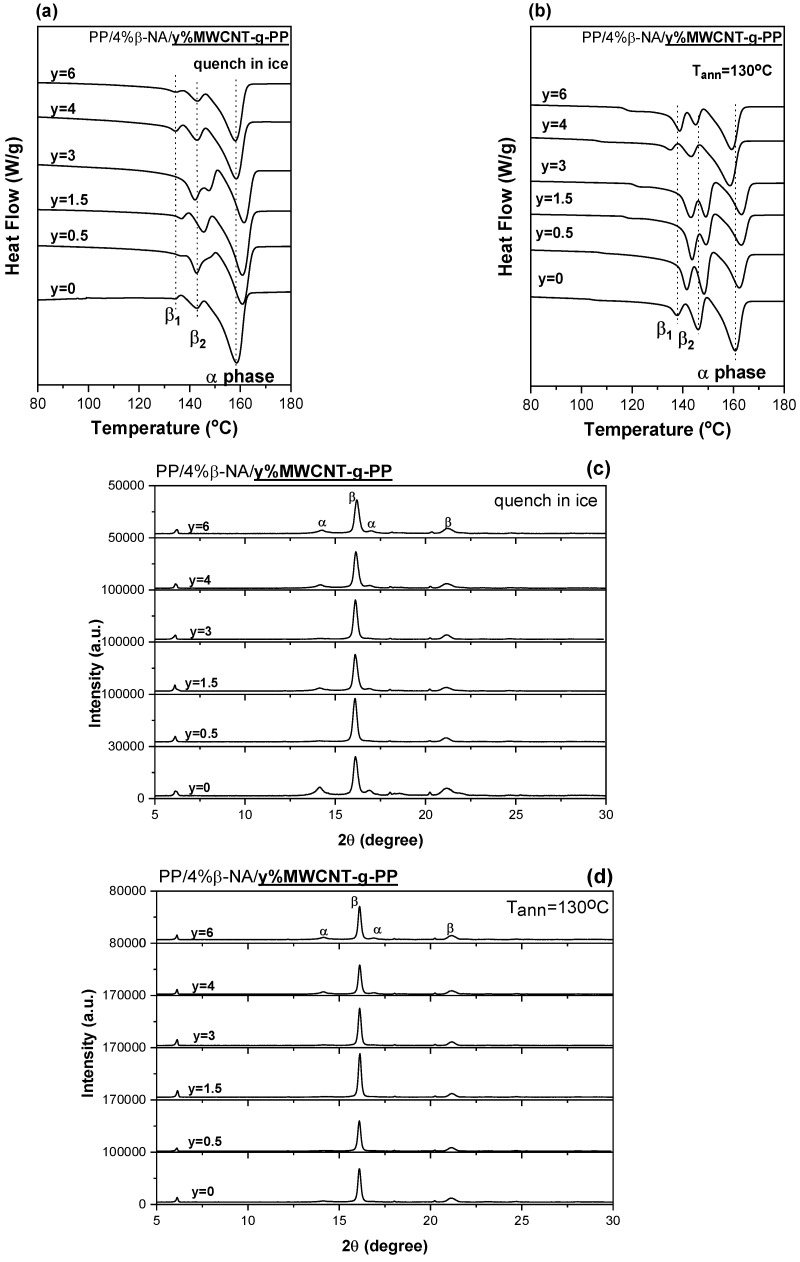
DSC thermographs of PP/4%β-NA/MWCNT-g-PP (0, 0.5, 1.5, 3, 4 and 6 wt.%) composite films (**a**) quenched in ice water from melt and (**b**) annealed at 130 °C); (**c**,**d**) XRD patterns of the corresponding composite films.

Samples containing different wt.% of nucleating agent with varying wt.% of carbon nanotubes were prepared. Here, we present results of PP composites bearing 4 wt.% of β-ΝA with 0.5, 1.5, 3, 4 and 6 wt.% of MWCNT-g-PP. Similar studies were performed for β-NA loadings of 0.3% and 1% and the results are depicted in the Supplementary Information (Figures S2 and S3 and Tables S2 and S3). From the DSC thermographs of PP/4%β-NA/0.5, 1.5, 3, 4 and 6% MWCNT-g-PP composite films, it is observed that apart from the melting peak of the α-phase of PP (~160 °C), the two characteristic peaks of the PP β-phase appear (β1~138 °C and β2~145 °C), which are particularly enhanced in the composite films crystallized at 130 °C (Figure 3b). This was also verified by the XRD patterns shown in Figure 3d. Obviously, the introduction of both β-NA and CNTs with efficient β- and α- nucleating ability, respectively, in PP, may induce a competitive growth between α- and β- modification. The values of crystallinity of either α or β phases obtained from the DSC thermographs are presented in Table 4 and depicted in Figure 3. In parenthesis are also presented the crystallinity values corresponding to the β-phase obtained from XRD measurements based in equations (1)-(3), shown in the Supplementary Information. A similar trend is followed for the β-crystalline values calculated from both methods. However, higher values are obtained from XRD measurements (SI).

The DSC plots show that upon incorporation of MWCNTs the melting peaks, either of β- or α-phase, are slightly shifted to higher temperatures up to 3 wt.%. MWCNT-g-PP, which indicates that the crystals became more perfect or bulkier due also to the intermolecular cross-linking action of CNTs specifically for α-phase crystals. However, further increase in the composition of MWCNTs (>4 wt.%) shifted the melting peaks back to the temperatures corresponding to the composite containing only β-NA. This could be explained by the fact that once a MWCNTs concentration threshold is reached, mostly due to their pronounced aggregation, PP polymer chain is constrained and crystal growth is hindered. Furthermore, the values of Χβ% presented in Table 4 indicates that for the composite materials that contain 4 wt.% of β-NA and were subjected to annealing, the percentage of the β-phase remains relatively constant. In other words, the presence of β-NA seems to stabilize the β-phase in the composite. This stability is observed even when different amounts of MWCNTs are added to the composite, up to a loading of 4 wt.% of MWCNTs. However, as more MWCNTs are incorporated into the composite beyond 4 wt.%, there is a decrease in the percentage of the β-crystalline phase. This means that the presence of higher concentrations of MWCNTs has an adverse effect on the β-phase growth in the composite. The XRD patterns of these composites support the observed trend. Specifically, for composites with the same composition of β-NA, the β crystal content increases with the addition of only 0.5 wt. % of MWCNTs. However, when concentrations of 4 wt.% and above MWCNTs are added, the β crystal content decreases, as confirmed by the XRD patterns. 

Recalling the graphs of Figure 3, there is an impression that the β- phase content is underestimated in the DSC thermograms when compared with their fingerprints in the XRD diagrams. The relative content of β-form crystal, was evaluated using Turner-Jones (see SI) criterion [32,61].

The statement highlights the importance of considering the effect of β-α recrystallization in the accurate determination of polymorphic composition when using calorimetric studies, specifically, differential scanning calorimetry (DSC) as Varga et al. demonstrated [36,37,38,39,62]. This recrystallization phenomenon occurs during the heating run process of samples. When studying the melting behaviour of PP, the presence of a nucleating agent can lead to the observation of a smaller or larger melting peak of the α-phase. This means that during the endothermic melting observed by DSC measurements of PP samples containing nucleating agent, the melting peak of the α-phase is overestimated from its true value in the original, pre-DSC sample. In conclusion, special care must be taken when drawing conclusions from calorimetric measurements performed on such samples. In particular, when estimating the amount of the α-phase and its melting temperature based on the analysis of melting peaks, it is important to critically evaluate the results. This is beyond the scope of the present work where the main focus is on the water vapour permeability behavior by the addition of fillers to PP films. In any case, for the elucidation of this β to α recrystallization occurring during the DSC heating run process, temperature dependent XRD measurements are presented in the Supplementary Information (Figure S5). According to the results obtained, the amount of α-phase in PP samples loaded with β-NA, increases at expenses of the β-phase when samples overpass T = 140 °C, while this change has already started from even lower temperatures 120–130 °C. At the same manner, DSC melting curves of isothermally crystallized samples (annealed at 130 °C) and submitted to T = 152 °C (which is the offset melting of the β-phase) are shown in Figure S6 corroborating this β to α recrystallization.

### 4.2. Water Vapor Transport of Hybrid PP/β-ΝA/MWCNT-g-PP Films

**Figure 4:**
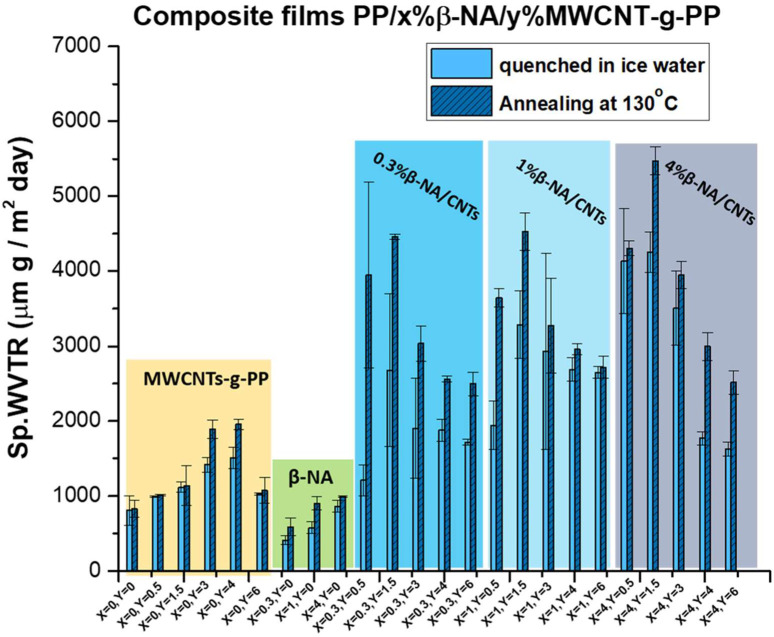
Specific water vapor permeability values, Sp.WVTR, for annealed (130 °C) and quenched composite films PP/x%β-NA/y%MWCNT-g-PP.

The main scope of this study is to evaluate whether the simultaneous addition of β-ΝA and MWCNT-g-PP could contribute to increase the water vapor permeability of PP films, and to establish the conditions that promote the best performance. It should be further noted that the hybrid films were prepared without the addition of CaCO_3_ (or other similar fillers) and without stretching of the films, the current industrial energy-consuming benchmark to increase the porosity of PP membranes. In this context, the water vapor permeability values for the annealed and quenched hybrid films (PP/x%β-NA/y%MWCNT-g-PP) measured at 27 °C and 21% relative humidity (RH) by the wet cup method are presented in Figure 4.

The first clear observation is that for polypropylene composite films containing only MWCNTs-g-PP, the water vapor permeability increases up to a critical filler concentration of 3–4 wt.% where the SpWVTR exhibits a maximum in the order of 2000 μm g/m^2^.day and decreases again at higher filler concentrations. This decrease has already been attributed either to the formation of aggregates of the nanofillers and/or to the formation of CNTs labyrinth-like networks that act as traps for migrating water [17]. 

The composite films annealed at 130 °C showed somewhat higher water vapor permeability values compared to those quenched in ice water, but without any noticeable difference.

In the case of polypropylene composite films containing only β-NA, for the range of concentrations used, the composition that gives the optimal WVTR (of around 1000 μm g/m² day) is that of the maximum concentration, 4 wt.%; it is worth noticeable that no water vapor permeability is detected in the master batch of PP/10 wt.% β-NA (Figure S4 and Table S4). Furthermore, the WVTR values observed for these films are in the range of the WVTR values obtained for neat PP; most probably, additional deformation (i.e., via uniaxial stretching) is needed [49] for the development of microporous membranes with high(er) porosity in PP/β-NA films.

In the case of polypropylene composite films, produced by melt mixing β-NA and MWCNT-g-PP with i-PP in a twin-screw extruder and subsequent melt press, a significant enhancement of WVTR is observed. The synergistic action of both fillers seems to play an important role in the development of effective porosity for water vapor permeability, which the presence of the highly water vapor permeable CNTs could further facilitate. The pore formation mechanism produced during the stretching of β nucleated polypropylene films has been widely analyzed in literature [50] and is explained by the fact that these micropores are directly created at the weak areas or interfaces between β-lamellae and enlarge with further deformation. In the case of cold drawing, a β- to α- solid state transformation could be envisaged and since polypropylene α-crystal exhibit a higher density (0.946 g/cm^3^) in comparison to the β-crystal (0.921 g/cm^3^) [63], further voids could be formed facilitating eventual WVT. 

In the present work, no external stretching was applied to the produced films. However, an increase in breathability has been observed when the two fillers, β-NA and carbon nanotubes, are simultaneously added to PP and used synergistically to avoid the stretching step. The stress here can be considered to be exerted on the essentially weak interfaces between the β-lamellas by the vigorous MWCNTs-induced growth of neighboring α-phase crystals and thus the composite microporosity is enlarging. At the same time, the presence of the frictionless graphenic walls of CNTs in the proximity of these enlarged micropores can reveal obvious tortuous pathways and facilitate water vapor permeability. Also noteworthy is the fact that the maximum WVP measured for the PP/β-NA/MWCNT-g-PP hybrid films were that at 1.5 wt.% MWCNTs for all b-NA loadings attempted. There is again a max in the WVP in polymeric membranes with the CNTs loading. The increase of the concentration of MWCNTs from and beyond 4 wt.% most probably favors their agglomeration, negatively affecting the growth of both crystalline phases of PP and especially that of the β-one as well as the WVT through the respective membranes.

Beyond the growth of the β crystalline phase that creates micropores at the expense of α phase due to the abundant weak interfaces between β-lamellas, we could further anticipate that even a low degree of a β- to α- phase change in the vicinity of the CNTs can effectively create microporous voids in the polymer matrix. However, it is not easy to support such a scenario that would have to take into account a β- to α- solid transformation or melting and recrystallization.

### 4.3. Cytotoxicity Evaluation of CNTs

The amine-functionalized MWCNTs, MWCNT-NH_2_, and the chemically functionalized MWCNT-g-PP that have been used for the preparation of PP-based composites, have been investigated in terms of cytotoxicity. This has been accomplished given the application of these membranes and the possibility (if any) of release of the CNTs. Thus, the information, obtained is of great importance for the safe application of the CNTs-polypropylene based films. The safe concentration levels of these CNTs have been determined using the lung cancer line A549 since toxicity of CNTs is mainly attributable to their similar structures to asbestos known to trigger asbestosis, lung cancer and pleural malignant mesothelioma. At first, we evaluated the effects of MWCNT-NH_2_ and MWCNT-g-PP on A549 cell viability for 24 h and 72 h, as to determine the short- and long-term effects, respectively. Mitochondrial activity of A549 cells was monitored by treatment with 500, 200, 50, 10 and 1 μg/mL of MWCNT-NH_2_ and MWCNT-g-PP and MTT assay. 

**Figure 5:**
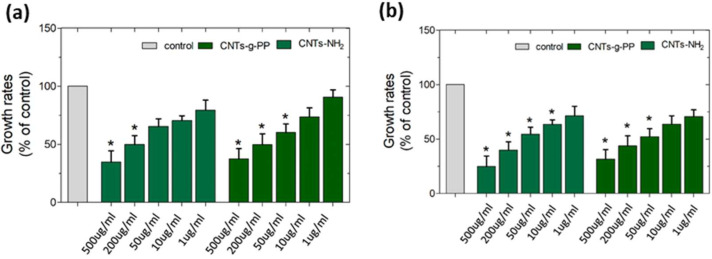
Effects of MWCNT-NH_2_ and MWCNT-g-PP on cell viability. Cell viability following 24 h (**a**) and 72 h (**b**) treatment of A549 cells with 1, 10, 50, 200, and 500 ug/mL of each MWCNT was assessed with MTT assay. Asterisk (*) indicates statistically significant differences (*p* < 0.05).

As depicted in Figure 5, both MWCNT-NH_2_ and MWCNT-g-PP demonstrated dose-dependent cytotoxicity both at short- and long-term treatment and a similar pattern on cell viability effects, while they exhibited no significant cytotoxic effects. Specifically, the most profound effects of MWCNT-NH_2_ on A549 cell viability at 24 h treatment (Figure 5a) was observed with 200 and 500 μg/mL of the amine-functionalized MWCNTs. However, in the lower concentrations tested, the viability levels were decreased only at 25–20% as compared to control (untreated) cells. As for the long-term effects (Figure 5b), MWCNT-NH_2_ 1–50 μg/mL did not significantly affect cell viability, as the maximum decrease in cell viability was 60% at 50 μg/mL as compared to control cells. Regarding the chemically functionalized MWCNT-g-PP, it demonstrated similar effects as MWCNT-NH_2_ both at 24 h (Figure 5a) and 72 h (Figure 5b) of treatment. Similarly, the effects on A549 cell viability followed a dose-dependent manner, whereas the decrease did not reach lower than 60% when cells were treated with up to 50 μg/mL as compared to the untreated cells. 

**Figure 6:**
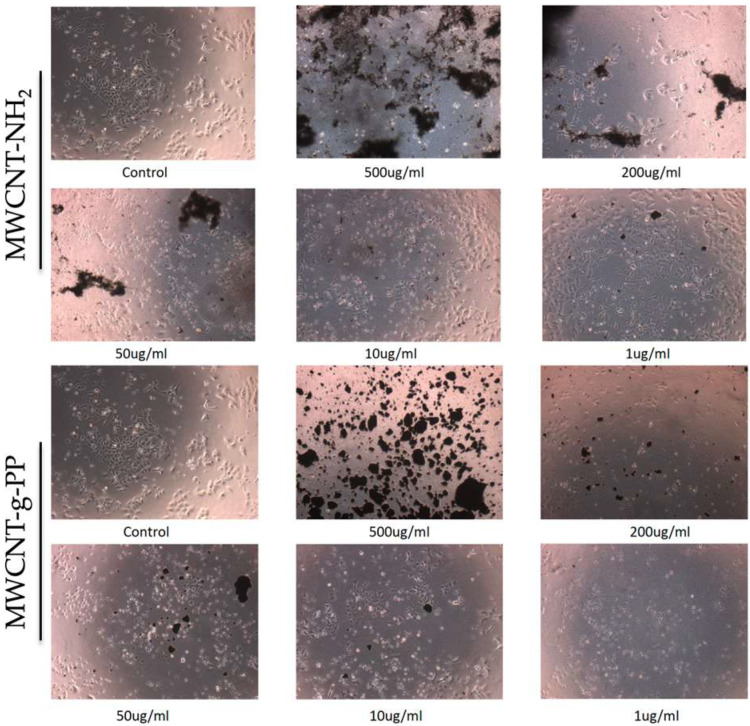
Phase contrast images with crystal violet dye of A549 cells cultured on pre-coated cell culture plates with 1–500 ug/mL MWCNTs.

At a next level, we evaluated the effects on cell morphology of A549 cells treated with MWCNT-NH_2_ and MWCNT-g-PP for 72 h as to determine the long-term effects. Both MWCNT-NH_2_ and MWCNT-g-PP demonstrated slight changes in cell cytoskeleton only at the highest concentration, compared with the control cells (Figure 6). As shown in Figure 6, no changes were found in cell morphology following treatment with MWCNT-NH_2_, while only in high concentration (200, 500 μg/mL) of both functionalized MWCNTs, slight changes in cell morphology were observed. Notably, the surfactant PF-127 did not show a statistically significant cytotoxicity in neither of the time-points tested and no morphological changes as well.

Taking into consideration the above data, we may suggest that in general, 1–50 μg/mL is considered a “safe” working concentration range of both MWCNT-NH_2_ and MWCNT-g-PP.

## 5. Conclusions

PP films containing different MWCNT-g-PP and β-NA loadings have been prepared and studied for their crystallization behavior (DSC and XRD) and water vapor permeability performance. Although MWCNTs act as an α-phase crystalline phase promoter for PP, the addition of even a small amount of β-NA (0.3 wt.%) allows the development of the crystalline β-phase particularly upon annealing at around 130 °C. Composites containing both, MWCNT-g-PP and β- nucleating agent, exhibited enhanced water vapor permeability than composites containing only one of the fillers. In addition, for these composites, thermal annealing seems to be more effective for increasing the β-phase and the WVTR. The presence of β-NA stabilizes the β-phase in the composite, while the incorporation of MWCNTs higher than 3 wt.% has a detrimental effect on the β-crystalline phase content, leading to a decrease in its percentage. There is, however, a threshold in what refers to MWCNT-g-PP loading with a maximum in the specific water vapor permeability that corresponds to 1.5 wt.% MWCNT-g-PP loading. 

In this context, polypropylene composite films exhibiting a specific WVTR up to 5500 μm·g/m^2^·day were produced taking advantage of the microporosity developed by the simultaneous incorporation of an α- and a β-nucletating agent as well as from the intrinsic water vapor transmitivity of the CNTs. The coexistence of a nanofiller, such as MWCNT-g-PP with inherent water vapor permeation properties, and a β-nucleating agent induced the competition of the growth between α- and β-crystal in the iPP matrix and an enhanced WVT. 

Parameters such as crystal growth mechanisms, specific heat treatment and type α- and β- NA, among others, should be considered in future works to optimize the contribution of both β-NAs and especially of graphene carbon allotropes to the WVTR of this new type of breathable membranes.

In respect to the potential risks and fates of MWCNTs, we demonstrated that both MWCNT-NH_2_ and MWCNT-g-PP demonstrated dose-dependent cytotoxicity in terms of cell viability, while they exhibited no significant effects in cell morphology after long-term exposure.

## Figures and Tables

**Table 1:** List of samples prepared during this study with PP as polymer matrix.

PP Composite Samples		
MWCNT-g-PP Loading (wt.%)	β-ΝA Loading (wt.%)	β-ΝA and MWCNT-g-PP Loading
0.5, 1.5, 3, 4, 6	0.3, 1, 4	All possible combinations

**Table 2:** Crystallinity values of composite films PP/MWCNT-g-PP from DSC thermographs.

Film	Χ_α_%	
	Samples Quenched	Samples Annealed 130 °C
PP	48.0	54.3
0.5% MWCNT-g-PP	39.3	43.7
1.5% MWCNT-g-PP	44.0	50.0
3% MWCNT-g-PP	49.0	50.4
4% MWCNT-g-PP	38.3	44.5
6% MWCNT-g-PP	40.5	44.7

**Table 3:** Crystallinity values of i-PP/(0.3, 1 and 4 wt.%) β-NA films from DSC thermographs.

Film	Thermal History	Χ_β_%	Χ_α_%	Χ%
PP	Tann = 130 °C		54.3	54.3
	quench in ice		48.0	48.0
0.3% β-NA	Tann = 130 °C	26.0	37.0	63.0
	quench in ice	11.0	34.0	45.0
1% β-NA	Tann = 130 °C	26.7	30.0	56.7
	quench in ice	11.0	31.7	42.7
4% β-NA	Tann = 130 °C	29.6	30.7	60.3
	quench in ice	21.3	36.0	57.3

**Table 4:** Crystallinity values of composite films PP/4%β-NA/y%MWCNT-g-PP from DSC thermographs. In parenthesis are listed the β-crystallinity (xβ) values for the annealed samples obtained from XRD measurements.

Film	Thermal History	Χ_β_%	Χ_α_%	Χ%
y = 0	Tann = 130 °C	29.6 (38)	30.7	60.3
	quench in ice	21.3	36.0	57.3
y = 0.5	Tann = 130 °C	31.7 (49)	19.0	50.6
	quench in ice	13.7	27.4	41.0
y = 1.5	Tann = 130 °C	33.6 (55)	17.4	51.0
	quench in ice	16.8	33.0	49.8
y = 3	Tann = 130 °C	31.6 (55)	17.9	49.5
	quench in ice	25.8	26.0	51.8
y = 4	Tann = 130 °C	17.4 (36)	36.0	53.4
	quench in ice	9.7	38.2	47.9
y = 6	Tann = 130 °C	17.4 (36)	31.9	49.3
	quench in ice	4.7	36.5	41.2

## Data Availability

The raw data will be available from corresponding author upon reasonable request.

## Supplementary Materials

The following supporting information can be downloaded at: https://www.mdpi.com/article/10.3390/polym15224432/s1. Equations used to evaluate the specific water vapor permeability. Figure S1: SEM images of a cryogenic cross-section fracture of: (a,b) PP/0.5%MWCNT-g-PP and (c–e) PP/4%MWCNT-g-PP showing agglomeration of MWCNTs in several magnifications. Figure S2: DSC thermographs of composite films PP/0.3%β-NA/MWCNT-g-PP (0, 0.5, 1.5, 3, 4 and 6 wt.%) (a) quenched in ice water from melt and (b) annealed at 130 °C); (c,d) XRD patterns. Table S1: Crystallinity values of composite films PP/0.3%β-NA/y%MWCNT-g-PP from DSC thermographs. Figure S3: DSC thermographs of composite films PP/1%β-NA/MWCNT-g-PP (0, 0.5, 1.5, 3, 4 and 6 wt.%) (a) quenched in ice water from melt and (b) annealed at 130 °C, (c,d) XRD patterns of the composite films. Table S2: Crystallinity values of composite films PP/1%β-NA/y%MWCNT-g-PP from DSC thermographs. Figure S4: DSC thermographs of PP/10%β-NA/ composite films quenched in ice water from melt and annealed at 130 °C (left) and XRD patterns of the composite films (right). Table S3: Crystallinity values of composite films PP/10%β-NA from DSC thermographs. Figure S5: XRD patterns of composite film PP/4%β-NA/3%MWCNT-g-PP as a function of temperature. Figure S6: Effect of the temperature on the DSC melting of composite film PP/4%β-NA/3%MWCNT-g-PP sample.
