# Supplementary material for: Improvement of Water Vapor Permeability in Polypropylene Composite Films by the Synergy of Carbon Nanotubes and β-Nucleating Agents
Source: Polymers (Basel). 2023 Nov 16;15(22):4432. doi: 10.3390/polym15224432 (PMC10674806; doi:10.3390/polym15224432)
Supplement: Supplementary file 1 [file polymers-15-04432-s001.zip › polymers-2665696-supplementary.pdf]

Supplementary Material

Improvement of Water Vapor Permeability in Polypropylene Composite Films by the Synergy of Carbon Nanotubes and β-Nucleating Agents

Glykeria A. Visvini [1,2], Georgios N. Mathioudakis [1,3], Amaia Soto Beobide [1], Zoi Piperigkou [4], Aris E. Giannakas [5], Stavros Messaritakis [6], Giannis Sotiriou [7] and George A. Voyiatzis [1,*]

[1] Foundation for Research and Technology-Hellas (FORTH), Institute of Chemical Engineering Sciences (ICE-HT), Stadiou Str., 265 04 Rio-Patras, Greece; gvisvini@iceht.forth.gr (G.A.V.); mathioy@iceht.forth.gr (G.N.M.), asoto@iceht.forth.gr (A.S.B.)
[2] Department of Physics, University of Patras, 265 04, Rio-Patras, Greece
[3] Department of Materials Science, University of Patras, 265 04 Rio-Patras, Greece
[4] Laboratory of Biochemistry, Department of Chemistry, Biochemical Analysis & Matrix Pathobiology Research Group University of Patras, 265 04 Rio-Patras, Greece; zoipip@upatras.gr
[5] Department of Food Science & Technology, University of Patras, 301 00 Agrinio, Greece; agiannakas@upatras.gr
[6] Plastika Kritis S.A., Industrial Area of Heraklion, R Street, Heraklion, 714 08 Crete, Greece; messaritakis@plastikakritis.com
[7] Thrace Polyfilms S.A., Industrial Area Xanthi, 67100 Xanthi, Greece; gsotiriou@thraceplastics.gr
* Correspondence: gvog@iceht.forth.gr.; Tel.: +30-2610-965253

Equations for WVTR calculation.

$$\mathrm{WVTR} = \frac{\text{water mass lost}}{\text{time x area}} = \frac{\text{flux}}{\text{area}}$$

$$\text{Sp.WVTR} = \text{WVTR} * l$$

Where area refers to the area of the film under measurement and l is the film thickness.

The wet cup measurements were carried out at Temperature of 27 °C, under humidity conditions of R_1=100% and R_2=21%.

The morphology of the polymeric composites was determined by scanning electron microscopy (SEM). A Zeiss ZUPRA 35 VP-FEG instrument, operating at 5−20 keV, was used.

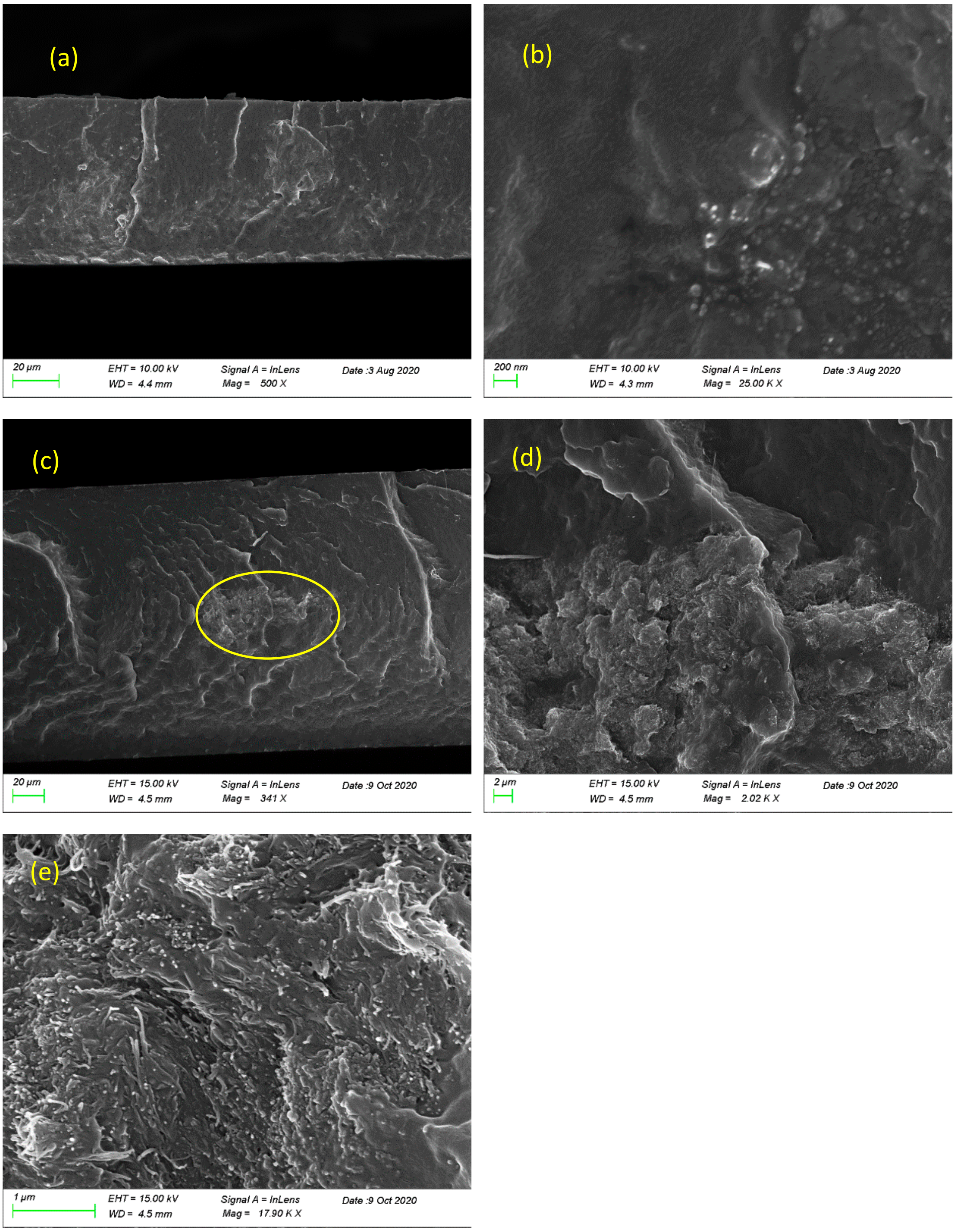

Figure S1: SEM images of a cryogenic cross-section fracture of: (a & b) PP/0.5%MWCNT-g-PP and (c,d & e) PP/4%MWCNT-g-PP showing agglomeration of MWCNTs in several magnifications. Areas with agglomeration are indicated with a yellow cicle.

The relative content of β-form crystal, was evaluated using Turner-Jones criterion as follows [32,61]:

$$x_\beta = K_\beta X_{all} \quad (1)$$

$$K_\beta = \frac{A_{\beta(300)}}{A_{\beta(300)}+A_{\alpha(110)}+A_{\alpha(040)}+A_{\alpha(130)}} \quad (2)$$

$$x_{all} = 1 - \frac{A_{amorphous}}{\sum A_{amorphous}+A_{crystalline}} \quad (3)$$

where $A\beta(300)$ represents the area of the (300) reflection peak, whereas $A\alpha(110)$, $A\alpha(040)$, and $A\alpha(130)$ represent the areas of the (110), (040), (130) reflection peaks, respectively. $A_{amorphous}$ and $A_{crystalline}$ are the areas of amorphous and crystalline peaks while xall and xβ are the overall crystallinity and crystallinity of the β-phase. The values Kβ obtained for samples annealed at 130 ^oC reach as high values as ~ 0,90 for carbon nanotubes loadings up to 3 wt%, however, this value achieves ~0.70 at higher MWCNTs loadings. The β-crystallinity (xβ) values for the annealed hybrid composites are listed in table 4 in parenthesis for comparison with the values obtained from DSC. A similar trend is followed for the β-crystalline values calculated from both methods, however higher values are obtained from XRD measurements. It is important to stress that the values derived from XRD cannot be directly compared to the ones by DSC. To assess crystallinity accurately, it is imperative to segregate the amorphous and crystalline components in the diffraction data. However, in this study, distinguishing between the amorphous halo and the prominent crystalline peaks is challenging due to the heightened intensity of the β-phase peaks.

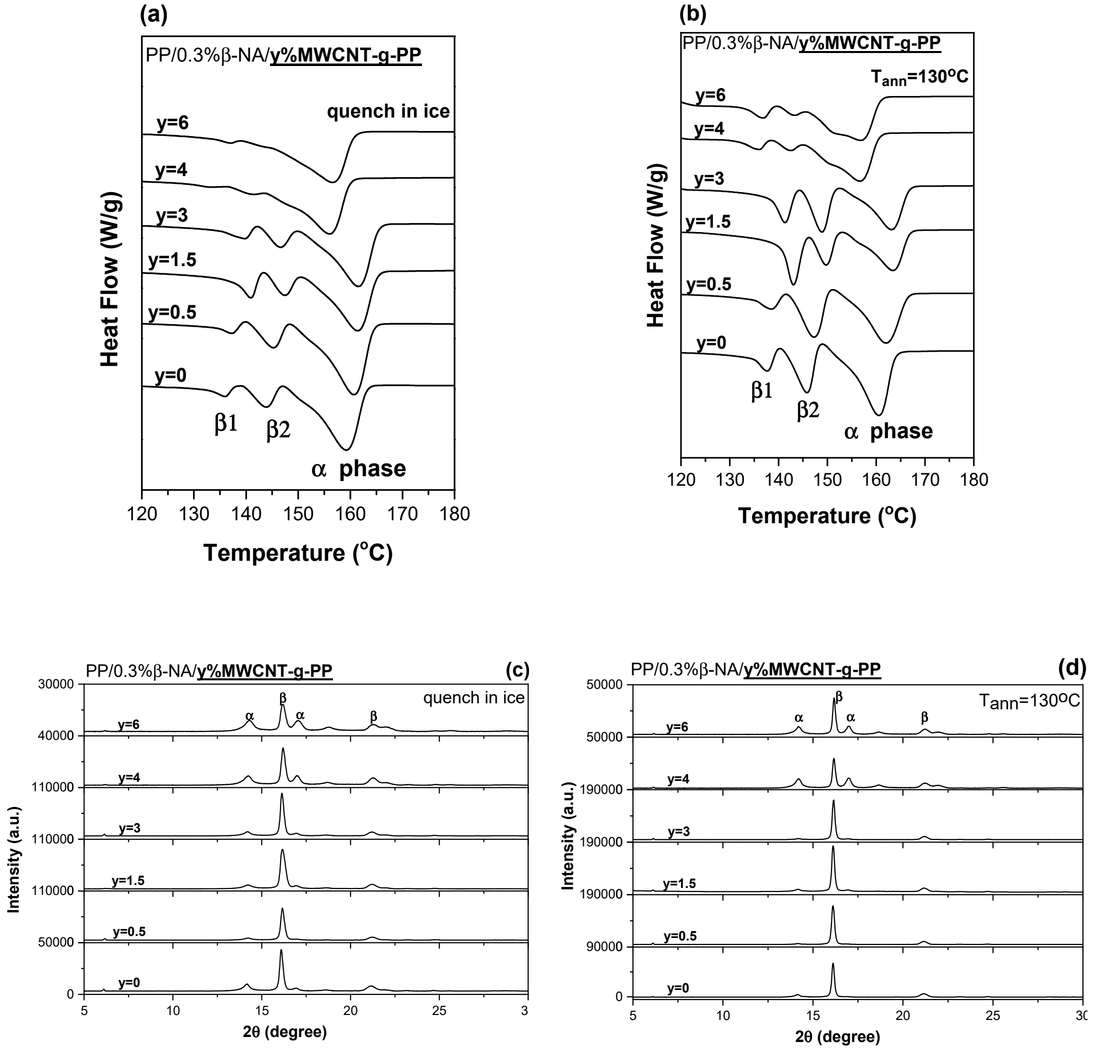

Figure S2. DSC thermographs of PP/0.3%β-NA/MWCNT-g-PP (0, 0.5, 1.5, 3, 4 and 6%wt) composite films (a) quenched in ice water from melt and (b) annealed at 130 °C); (c, d) XRD patterns of the composite films.

Table S1: Crystallinity values of composite films PP/0.3%β-NA/y%MWCNT-g-PP from DSC thermographs

Film	Thermal history	X_β%	X_α%	X%
y=0	Tann=130°C	26.2	37.0	63.2
	quench in ice	11.0	34.2	45.2
y=0.5	Tann=130°C	32.0	25.8	57.8
	quench in ice	16.4	39.0	55.4
y=1.5	Tann=130°C	20.0	24.2	44.2
	quench in ice	17.7	34.5	52.2
y=3	Tann=130°C	27.4	24.3	51.7
	quench in ice	15.1	37.0	52.1
y=4	Tann=130°C	6.5	36.1	42.6
	quench in ice	3.8	34.8	38.6
y=6	Tann=130°C	6.5	37.1	43.6
	quench in ice	1.0	39.5	40.5

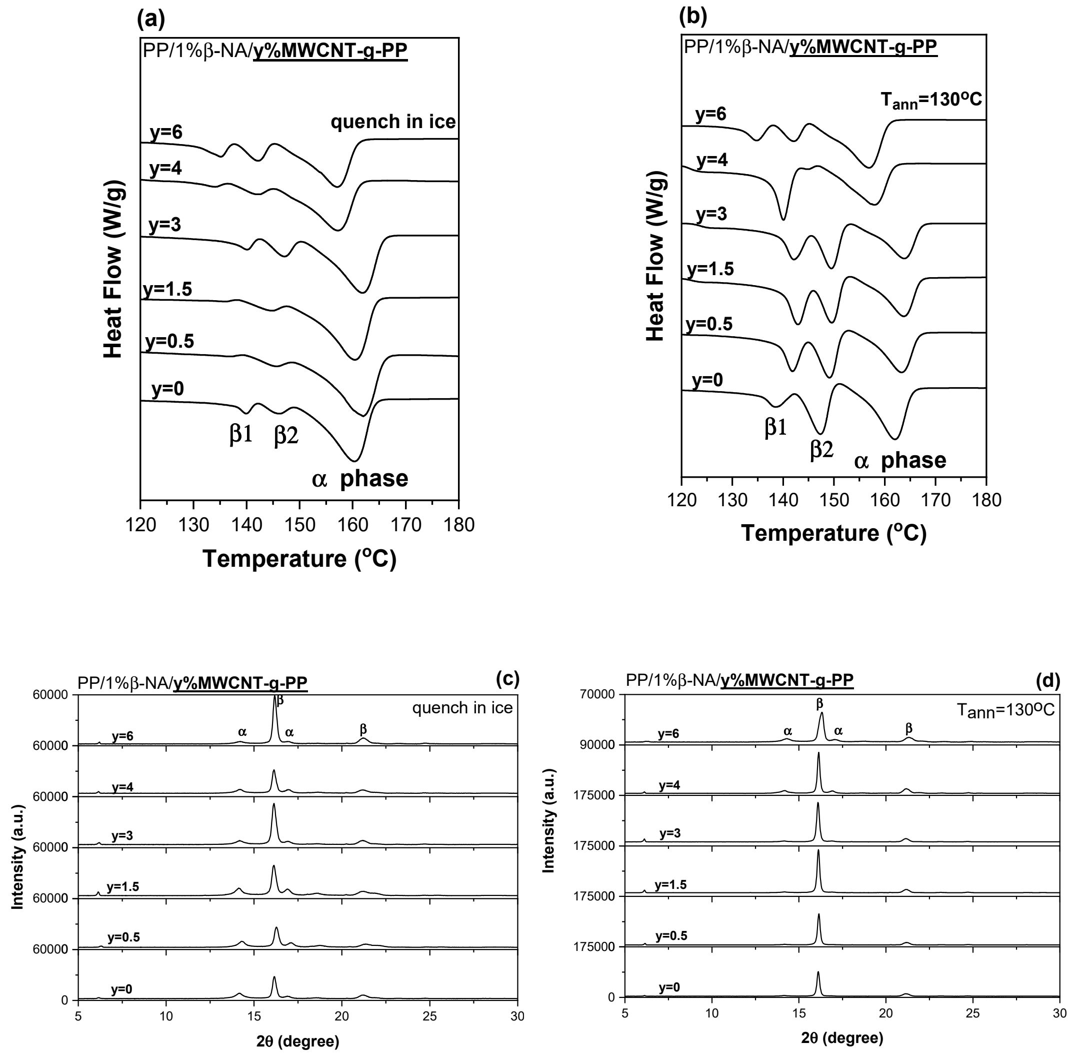

Figure S3. DSC thermographs of PP/1%β-NA/MWCNT-g-PP (0, 0.5, 1.5, 3, 4 and 6%wt) composite films (a) quenched in ice water from melt and (b) annealed at 130 ^{o}C); (c, d) XRD patterns of the composite films.

Table S2: Crystallinity values of composite films PP/1%β-NA/y%MWCNT-g-PP from DSC thermographs

Film	Thermal history	X_β%	X_α%	X%
y=0	Tann=130°C	26.7	30.0	56.7
	quench in ice	11.0	31.7	42.7
y=0.5	Tann=130°C	28.4	22.1	50.5
	quench in ice	4.3	38.6	42.9
y=1.5	Tann=130°C	28.2	23.8	52.0
	quench in ice	9.9	41.4	51.3
y=3	Tann=130°C	27.9	21.4	49.3
	quench in ice	13.7	37.6	51.3
y=4	Tann=130oC	14.7	30.0	44.7
	quench in ice	6.3	36.8	43.1
y=6	Tann=130oC	13.3	33.1	46.4
	quench in ice	9.3	30.4	39.7

Samples containing 0.3 wt% of nucleating agent but varying weight percentages of carbon nanotubes were prepared. From the DSC thermographs of PP/0.3%β-NA/0.5, 1.5, 3, 4 and 6% MWCNT-g-PP composite films it is observed that, apart from the melting peak of the α-phase of PP (~160°C), the two characteristic peaks of the β-phase of PP appear (β_1~138°C and β_2~145°C), which are particularly enhanced in the composite films annealed at 130°C (Fig. S2 a, b). This was also verified by the XRD patterns shown in figure S3 c,d. The same observations were also captured in the PP/1%β-NA/0.5, 1.5, 3, 4 and 6% MWCNT-g-PP composite films (Fig. S3 a, b, c, d).

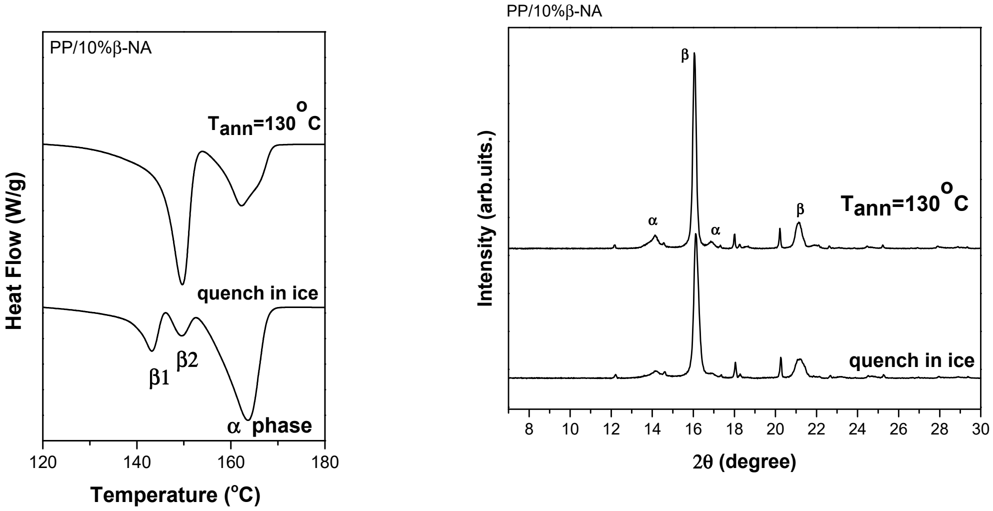

Figure S4. DSC thermographs of PP/10%β-NA/ composite films quenched in ice water from melt and annealed at 130 °C (left) and XRD patterns of the composite films (right).

Table S3: Crystallinity values of composite films PP/10%β-NA from DSC thermographs

Film	Thermal history	X_β%	X_α%	X%
10%	Tann=130°C	30.3	16.0	46.3
	quench in ice	9.0	30.2	39.2

For the verification of the β to α recrystallization occurring during the DSC heating run process concerning the overestimation of the α-crystalline phase in the accurate determination of polymorphic composition when using calorimetric DSC studies the following measurements were performed. A film with composition PP/4%β-NA/3%MWCNT-g-PP was submitted to temperature dependence XRD measurements and DSC thermal runs. The results are presented in Fig. S5 & S6.

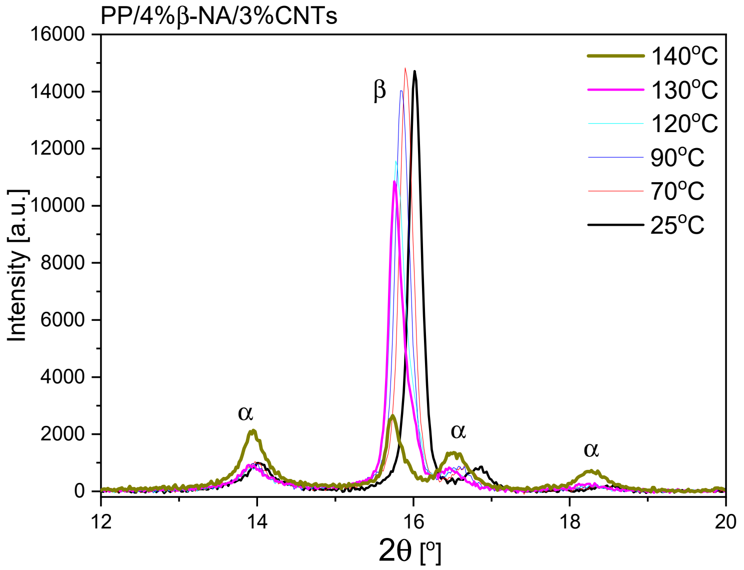

Figure S5: XRD patterns of composite film PP/4%β-NA/3%MWCNT-g-PP as a function of temperature.

Figure S5 displays the temperature dependence of the XRD of a PP/4%β-NA/3%MWCNT-g-PP film.

The film was subjected to a range of temperatures by placing it in the XRD, equipped with a XRK900 reactor chamber (Anton Paar GmbH). The temperature was increased to the desired one (10 min. were needed to reach each selected temperature) and the XRD diffractometer were recorded at each temperature.

It is observed that the refractive peak assigned to the β-phase remains constant from T=ambient to T=120°C. At higher temperatures, the melting or/and the recrystallization (to the α-one) of the β- crystalline phase is perceived by the decrease in the intensity of the peak at 16°; when the temperature reaches 140°C the peaks corresponding to the α-crystalline phase are enhanced at the expense of the β-refractive peak (dark yellow line). There is still a small peak at ~ 16° corresponding to β-crystalline phase because the $T_{offset\ (\beta)}$ = 150°C which is the temperature at which all the β-crystalline phase melts (see DSC below) has not been reached

This is also confirmed by the DSC measurements performed for the same PP/4%β-NA/3%MWCNT-g-PP film and displayed in Fig. S6

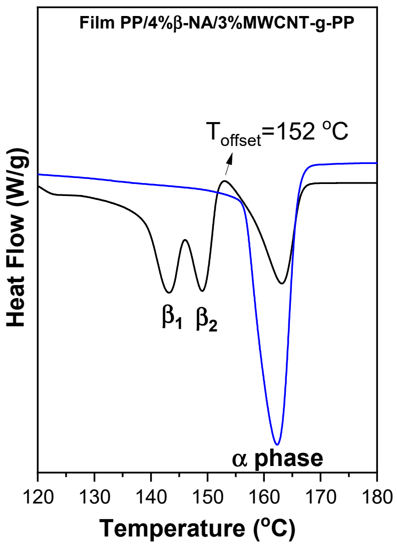

Figure S6: Effect of the temperature on the DSC meltings of composite film PP/4%β-NA/3%MWCNT-g-PP sample.

Figure S6 represents the melting curves of the PP/4%β-NA/3%MWCNT-g-PP sample crystallized at 130 °C where the α and β thermal melting points can be observed (black line). It is also presented (blue line) the melting curve of the same sample submitted to annealing at 152 °C which is the temperature corresponding to the offset of the β melting. It is clear that not only the peaks corresponding to the β crystalline phase have disappeared because they are melted but that the melting peak corresponding to the α-crystalline phase has increased.

The results obtained from figures S5&S6 corroborated the β to α recrystallization and care should be taken when conclusions regarding the percentage of α and β crystalline forms in PP are obtained from DSC measurements.
